# Design, conduct, and analysis of a multicenter, pharmacogenomic, biomarker study in matched patients with severe sepsis treated with or without drotrecogin Alfa (activated)

**DOI:** 10.1186/2110-5820-2-15

**Published:** 2012-06-13

**Authors:** Djillali Annane, Jean Paul Mira, Lorraine B Ware, Anthony C Gordon, Jonathan Sevransky, Frank Stüber, Patrick J Heagerty, Hugh F Wellman, Mauricio Neira, Alexandra DJ Mancini, James A Russell

**Affiliations:** 1 Service de reanimation medicale, CIC-IT805 (INSERM), Hopital R. Poincare (AP-HP), 104 Bd Raymond Poincare, 92380, Garches, France; 2 Université Paris Descartes, Sorbonne Paris Cité, Cochin Hotel-Dieu University Hospital Medical Intensive Care Unit, AP-HP, 75014, Paris, France; 3 Allergy, Pulmonary and Critical Care Medicine, Vanderbilt University School of Medicine, 1161 21st Avenue South T1218 MCN, Nashville, TN, 37232-2650, USA; 4 Section of Anaesthetics, Pain Medicine, and Intensive Care, Imperial College London, Charing Cross Hospital, Fulham Palace Road, London, W6 8RF, UK; 5 MICU, John Hopkins BMC, 5501 Hopkins Bayview Circle, Suite 4B-73, Baltimore, MD, 21224, USA; 6 Universitatsklinik fur Anasthesiologie und Schmerztherapie Inselspital, 3010, Bern, Switzerland; 7 Department of Biostatistics, University of Washington, F-600, Health Sciences Building, Box 357232, Office: H-665D HSB, Seattle, WA, 98195-7232, USA; 8 Sirius Genomics Inc, 603-1125 Howe St, Vancouver, BC, V6Z 2K8, Canada; 9 Critical Care Research Laboratories, The James Hogg iCAPTURE Centre for Cardiovascular and Pulmonary Research, St. Paul’s Hospital and University of British Columbia, Burrard Building, Rm 166-1081 Burrard St, Vancouver, BC, V6Z 1Y6, Canada; 10 Hospital Raymond Poincaré (AP-HP), University of Versailles SQY, 104 boulevard Raymond Poincaré, 92380, Garches, France

**Keywords:** Drotrecogin alfa (activated), Pharmacogenomics biomarker, Predictive marker, Propensity score, Severe sepsis, Treatment selection, Sepsis, Drotrecogin alfa activated (DAA), Activated protein C, Genome wide association study, Survival

## Abstract

**Background:**

A genomic biomarker identifying patients likely to benefit from drotrecogin alfa (activated) (DAA) may be clinically useful as a companion diagnostic. This trial was designed to validate biomarkers (improved response polymorphisms (IRPs)). Each IRP (A and B) contains two single nucleotide polymorphisms that were associated with a differential DAA treatment effect.

**Methods:**

DAA is typically given to younger patients with greater disease severity; therefore, a well-matched control group is critical to this multicenter, retrospective, controlled, outcome-blinded, genotype-blinded trial. Within each center, DAA-treated patients will be matched to controls treated within 24 months of each other taking into account age, APACHE II, cardiovascular, respiratory, renal, and hematologic dysfunction, mechanical ventilation status, medical/surgical status, and infection site. A propensity score will estimate the probability that a patient would have received DAA given their baseline characteristics. Two-phase data transfer will ensure unbiased selection of matched controls. The first transfer will be for eligibility and matching data and the second transfer for outcomes and genotypic data. The primary analysis will compare the effect of DAA in IRP + and IRP − groups on in-hospital mortality through day 28.

**Discussion:**

A design-based approach matching DAA-free to DAA-treated patients in a multicenter study of patients who have severe sepsis and high risk of death will directly compare control to DAA-treated groups for mortality by genotype. Results, which should be available in 2012, may help to identify the group of patients who would benefit from DAA and may provide a model for future investigation of sepsis therapies.

## Background

There are approximately 750,000 new cases of severe sepsis per year in the United States (US) [1] and 300,000 in the European Union (EU) [2]. The incidence of septic shock is increasing [3] and mortality from severe sepsis and septic shock is high, ranging from 30% to 60%, respectively.

PROWESS, the first Phase III, randomized, controlled trial of drotrecogin alfa (activated) (DAA) (recombinant human activated protein C), demonstrated an absolute risk reduction (ARR) of 6.1% in the 28-day mortality rate (*P* = 0.005) in severe sepsis [4]. The U.S. Food and Drug Administration (FDA) approved DAA but raised concerns, because the PROWESS protocol was amended approximately half way through the study, with modification of eligibility criteria, study objectives, and covariates for adjustment of the primary endpoint [5,6]. Other trials have not lessened the controversy about DAA. The ADDRESS study in sepsis patients at low risk of death [7], and the RESOLVE trial, in children with sepsis-induced cardiovascular and respiratory failure [8], were both stopped for futility. Moreover, there are safety concerns, including increased incidence of serious bleeding after DAA compared to placebo (5.6% DAA; 2.0% placebo, *P* < 0.001) [9].

The FDA approved DAA for patients with severe sepsis and high risk of death (e.g., APACHE II ≥ 25) [10]. Marketing authorization in the EU was granted for patients with severe sepsis and two or more organ failures. The EU approval was subject to annual reviews and the provision of additional data to support efficacy and safety [11]. PROWESS SHOCK, a randomized, placebo-controlled trial of DAA in 1,696 patients with septic shock [12] showed that 28-day mortality was 26.4% and 24.2% in the DAA and placebo arms, respectively (*P* = 0.31), with remarkably low rates of serious bleeding (1.2% vs. 1%). On October 25, 2011, Eli Lilly and Company withdrew DAA from the market worldwide. For PROWESS SHOCK, the observed pooled mortality was much lower than expected, lower than in PROWESS, which enrolled a broader population of severe sepsis [4]. The low mortality rates observed in PROWESS SHOCK may be explained in part by recent advances in the management of septic shock and in part by the selection of lower risk patients. If DAA is to be reintroduced clinically, an effective strategy must involve better tools for the selection of patients who will respond to DAA. The study design and statistical methods for this study have been discussed with the U.S. FDA.

### Background on selection of pharmacogenomic biomarkers for current study (SGX301)

Pharmacogenomic markers identify patients predicted to have increased efficacy or greater likelihood of adverse effects of many drugs [13]. To screen for genomic biomarkers, a Genome Wide Association Study (GWAS) of the PROWESS study was performed (unpublished data). The GWAS used blood spot samples from 1,446 patients to genotype approximately 1.2 million SNPs (Illumina® Human1M-Duo BeadChip). Findings were then tested in a small, combined replication cohort drawn from the single-center St. Paul’s Hospital (SPH) registry and the multicenter Vasopressin and Septic Shock Trial (VASST) [14].

The combined replication cohort of 738 patients had 141 patients treated with DAA. Baseline characteristics of DAA-treated and DAA-free patients showed significant differences: DAA-treated patients were younger and sicker. Because these imbalances could confound mortality assessments, matching of controls (up to three DAA-free patients for every DAA-treated patient) was done and achieved balance between groups (Table 1). This matching strategy will be used in our current study as described below.

**Table 1:** Baseline characteristics of replication cohorts before and after matching

	**Before matching**						**After matching**		
**Demographic or disease characteristic**	**VASST cohort**			**SPH cohort**			**VASST and SPH combined**		
	DrotAA(n = 103)	**Control**(n = 370)	***P*value**	**DrotAA**(n = 38)	**Control**(n = 227)	***P*value**	**DrotAA**(n = 130)	**Control**^a^ (n = 286)	***P*value**
**Age**									
Mean ± SD	57.6 ± 15.6	62 ± 15.4	0.009	54.6 ±20.1	61 ± 14.9	0.072	58.4 ± 15.4	58.7 ± 15.3	0.886
**Women** (percentage of patients)	35.9%	41.4%	0.32	44.7%	33.5%	0.178	40%	36.8%	0.596
**APACHE II score**									
Mean ± SD	27 ± 7.4	0.253	25.7 ± 6.4	22.9 ± 7.2	0.02	27.1 ± 5.8	27 ± 5.9	0.862	
**Medical** (percentage of patients)	85.4%	76.5%	0.051	89.5%	83.3%	0.332	86.2%	79.4%	0.106
**Organ failure** (percentage of patients)									
Cardiovascular	100%	100%	1	84.2%	82.4%	0.783	95.4%	96.2%	0.418
Respiratory	85.4%	85.4%	0.994	94.7%	93%	0.686	86.9%	86%	0.71
Renal	61.2%	46.2%	0.007	47.4%	46.3%	0.899	59.2%	55.5%	0.172
Hematologic	23.3%	19.5%	0.391	13.2%	13.7%	0.934	19.2%	15.1%	0.292
**Ventilation** (percentage of patients)	98.1%	93.5%	0.073	100%	89.4%	0.036	99.2%	99.2%	1
**Caucasian** (percentage of patients)	80.6%	83.2%	0.528	NA	NA		NA	NA	

^a^Weighted values for control group.

As recommended by international guidelines for association studies [15], the replication cohort was used to confirm individual SNP results. Two-SNP composite improved response polymorphisms (IRPs), A and B, were constructed. Patients were classified as IRP A + or − and IRP B + or − if they had one of both of the responsive genotype. The individual SNPs in each IRP were associated with a differential DAA treatment effect in the PROWESS study and replicated in the combined replication cohort (unpublished).

The two SNPs comprising IRP A were chosen based first on the alignment of direction and strength of their signals by analyzing the interaction of SNP and treatment effect on mortality in both the PROWESS study and the replication cohort. Secondly, these two SNPs were chosen based on the known biological plausibility linking these SNPs to underlying pathways of sepsis or pathways that could affect the mechanisms of action of DAA. The two SNPs comprising IRP A are RYR2 (ryanodine receptor 2 gene) rs684923 on chromosome 1 and ACIN1 (apoptotic chromatin condensation inducer 1 gene) rs3751501 on chromosome 14. The SNP of RYR2 could act to enhance efficacy of activated protein C on protection of endothelial permeability via its effects on endothelial protein C receptor and sphingosine-1-phosphate receptor 1 [16]. Phosphorylation of a residue (S422) inACIN1 (Acinus-S variant) by AKT (prosurvival kinase), completely inhibits cleavage of Acinus-S by caspase-3, abrogating the formation of fragment p17 which is essential for chromatin condensation during apoptosis [17]. As a result, phosphorylation of S422 by AKT is reduced by the lack of phosphorylation in amino acid residue S573. It is conceivable that lack of phosphorylation in S478 due to 478 F mutation and the change of polarity caused by the change from a polar amino acid (S) to a nonpolar amino acid (F) would greatly impact Acinus-S protein conformation and probably affect the likelihood of S422 phosphorylation by AKT. If this were the case, the genetic variants rs3751501 (AA|AG), associated with increased ARR (absolute risk reduction) and coding for amino acid 478 F in ACIN1, would render ACIN1 constitutively nonphosphorylated at residue 478 F and hence constitutively nonphosphorylated at S422, leading to AKT-independent regulation of chromatin condensation by Acinus-S during apoptosis, because nonphosphorylated acinus-S would be constitutively cleavable by caspase-3. In such a situation, cleavage of Acinus-Sby caspase-3 would be more sensitive to inhibitors of caspase-3, for example, inhibition of caspase-3 by rhAPC, which would be consistent with the genotype (rs3751501) by treatment interaction seen in our studies.

Two SNPs comprising IRP B were chosen based solely on the strength of their signals in PROWESS and in the replication cohort, without regard to biological plausibility. These two SNPs are SPATA7 (spermatogenesis associated 7 gene) rs3179969 on chromosome 14 and FLI1 (Friend leukemia virus integration 1 gene) rs640098 on chromosome 11.

**Figure 1:**
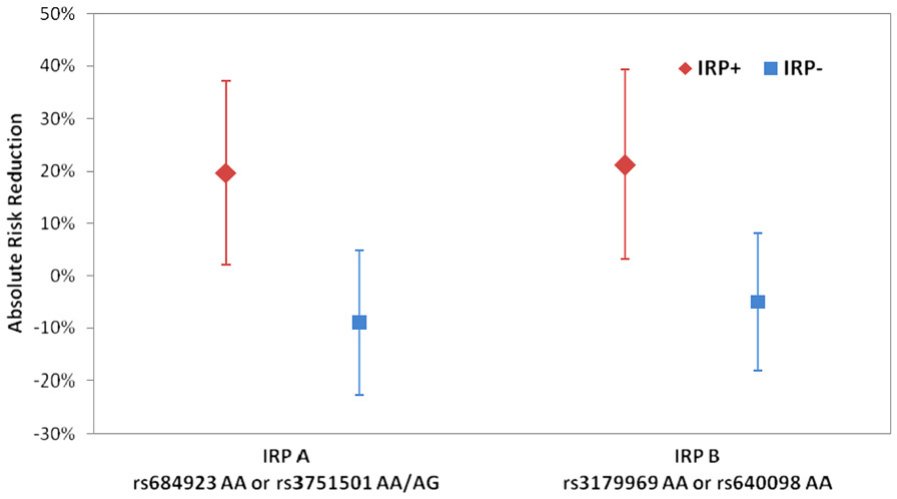
**ARR was 19.7% for IRP A + patients (95% CI 2.2–37.1%) and −8.9% for IRP A − patients (95% CI −22.6 to 4.9%).** The SNP-by-treatment interaction *P* value was 0.018 unadjusted and 0.066 adjusted for matching covariates. The proportion of patients who were IRP A + was 33.7% (140/415) in the replication cohort. The ARR was 21.2% for IRP B + patients (95% CI 3.2–39.2%) and −5% for IRP B − patients (95% CI −18.2 to 8.2%). The SNP-by-treatment interaction *P* value was 0.04 unadjusted and 0.069 adjusted for matching covariates. The proportion of patients who were IRP B + was 26.1% (107/410) in the replication cohort.

The ARR in mortality by IRP status in the combined replication cohort is shown in Figure 1. The ARR was 19.7% for IRP A + patients (95% confidence interval (CI) 2.2–37.1%), whereas for the IRP A − patients the ARR was −8.9% (95% CI −22.6 to 4.9%). The SNP-by-treatment interaction *P* value was 0.018 unadjusted and 0.066 adjusted for matching covariates. The proportion of patients who were IRP A + was 33.7% (140/415) in the replication cohort. The ARR was 21.2% for IRP B + patients (95% CI 3.2–39.2%), whereas for the IRP B − patients the ARR was −5% (95% CI −18.2 to 8.2%). The SNP-by-treatment interaction *P* value was 0.04 unadjusted and 0.069 adjusted for matching covariates. The proportion of patients who were IRP B + was 26.1% (107/410) in the replication cohort.

### The SGX301 study

#### *Hypothesis and overall design*

**Figure 2:**
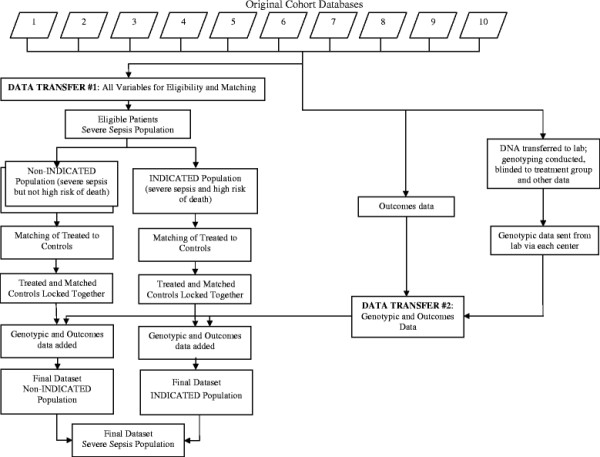
**In data transfer #1, data from each patient in each of the ten cohorts are submitted and patients are considered for eligibility criteria**. Then, patients are segregated into the non-INDICATED (do not meet criteria for high risk of death as per FDA and EU approvals for DAA) and INDICATED (fulfill FDA or EU approvals for DAA) populations (see text for details). Then, DAA-treated patients are matched with DAA-free patients (controls). Subsequently, the matched sets are locked together. Then, the genotype and outcomes data are transferred (data transfer #2 described below) for each patient thereby creating a final dataset of non-INDICATED and INDICATED populations. Finally, the non-INDICATED and INDICATED datasets are merged to create the final dataset of the severe sepsis (SEVSEP) population. In parallel with the matching process, each center sends DNA for genotyping to the laboratory and genotyping is done while blinded to treatment group and outcome. The genotype data and outcome data are then sent via each center to the central dataset as data transfer #2.

The study hypothesis is that IRP A and/or IRP B predict a differential DAA treatment effect in patients with severe sepsis and high risk of death. The design of this international, multicenter, retrospective, controlled, outcome-blinded, genotype-blinded, matched-patients study is depicted in Figure 2. Retrospectively collected DNA and clinical data will be analyzed to validate the prespecified IRPs. Some of the cohorts are drawn from patient registries and others are from clinical trials where the primary hypothesis was not related to DAA. Prospective aspects of this study are the genotyping of patients with regard to the IRPs and the statistical testing of the prespecified hypothesis regarding the interaction of IRP genotypes and DAA treatment on mortality. Eight academic centers will contribute data and DNA from ten cohorts (5 EU, 4 USA, 1 Canada).

For each IRP, individual patients will be considered to be biomarker positive if they have the responsive genotype for either of the SNPs or for both of the SNPs in the IRP.

### Ethics

All cohorts included in this study have complied with local requirements with respect to requiring written, informed consent and ethics committee oversight.

### Study population and treatment groups

To be included in the current study, patients must meet eligibility criteria for the INDICATED population and subsequently, DAA-treated patients will be matched to DAA-free patients. Eligibility criteria, consistent with the approved use of DAA in the United States and the European Union, will be used to select the primary study population (INDICATED) from among all patients enrolled in the ten contributing cohorts (Table 2). This population with high risk of mortality reflects common practice for current use of DAA [18-23]. A second study population with severe sepsis (non-INDICATED) also will be selected in which severe sepsis patients do not necessarily meet the high risk of death criteria. This population will include all patients who meet criteria 1, 2, 4, and 5 in Table 2 from the ten cohorts. The INDICATED and non-INDICATED populations will be merged to constitute the SEVSEP population (Figure 2).

**Table 2:** Eligibility criteria for INDICATED population

Eligibility criteria consistent with the approved use of DAA in the USA and the EU will define the INDICATED population as follows:	
1	Men or women age 18 years or older
2	Must have severe sepsis (must meet a, b, and c below)
a)	Suspected or proven infection
b)	Systemic inflammatory response syndrome (SIRS) (must meet 2 of 4 criteria)
i)	Temperature <36°C or >38°C
ii)	Heart rate >90 beats/minute
iii)	Respiratory rate >20 breaths/minute or PaC0_2_ <32 mm Hg or on mechanical ventilation
iv)	White blood cell count <4,000/mm^3^ or >12,000/mm^3^
a)	At least one organ dysfunction due to sepsis based on definitions of clinically significant organ dysfunction.
i)	Cardiovascular dysfunction [must meet one of (1), (2), or (3) below]:
(1)	systolic blood pressure ≤90 mmHg and pH ≤7.3
(2)	mean arterial pressure ≤70 mmHg and pH ≤7.3
(3)	reported use of a vasopressor alone is sufficient evidence of shock
i)	Pulmonary dysfunction: PaO_2_/FiO_2_ ≤300 mm Hg
ii)	Central Nervous System dysfunction: Glasgow Coma Score ≤12
iii)	Coagulation dysfunction: platelets ≤80,000/mm^3^
iv)	Renal dysfunction: creatinine ≥2.0 mg/dL
v)	Hepatic dysfunction: bilirubin ≥ 2.0 mg/dL
1)	High risk of death (one of a, b, or c below)
a)	APACHE II ≥25
b)	SAPS II ≥54
c)	Multiple organ dysfunction – two or more clinically significant organ dysfunctions (as defined above), which have occurred within 2 days of each other
2)	Platelet counts ≥30,000/mm^3^
3)	DAA status known

Patients at increased risk of bleeding due to low platelet counts are contraindicated for DAA use and will be excluded from the INDICATED population. An additional requirement for all patients will be that they had to have been treated for severe sepsis either after DAA was made available in their hospital (if known) or after the drug was approved in their country or within 24 months before that date. This will ensure that control (non-DAA–treated) patients are from a similar time period as the DAA-treated patients.

No patients will be prospectively treated under this protocol. DAA-treated patients were given DAA in accordance with local regulatory approvals and clinical practice in each center. The recommended regimen for DAA is 24 μg/kg per hour continuous intravenous infusions for 96 hours. The timing of DAA administration relative to day 1 (the day of diagnosis of severe sepsis with high risk of death) will be collected when available.

### Matching of DAA-treated to control patients

In practice, DAA is typically given to younger patients with greater disease severity. Therefore, a well-matched control group is critical to the validity of this nonrandomized study. Therefore, our study design incorporates an overall matching strategy. First, to control for differences in standard of care among centers and over time, DAA-treated patients will be matched to controls (DAA-free patients) enrolled within 2 years in the same cohort. After eligibility is met, control patients will be selected to match the DAA-treated patients using a computerized optimal matching algorithm matching baseline demographic and disease characteristics that have been identified *a priori* as likely influencing the decision to give DAA or the probability of death. The number of matched control patients for each DAA-treated patient will be variable (one to three). This strategy increases the precision in the estimation of the differences between groups [24,25].

The matching strategy will combine minimum-distance matching with the use of “calipers” that force the matches for selected variables to fall within specified tolerances. Individual variables will be used to compute a multivariate distance (Mahalanobis distance). A propensity score that is the estimated probability that a patient would have received DAA given their key baseline characteristics will be calculated. Rosenbaum and Rubin suggested that to obtain covariate balance, an approach combining both the propensity score and covariate matching is superior to the use of either strategy alone [26]. The intended clinical variables for the calculation of the Mahalanobis distance and the reasons these variables were chosen (based on literature review and discussions with coinvestigators) are in Table 3.

**Table 3:** Rationale for selected Mahalanobis distance variables

**Baseline characteristic(variable)**	**Associated with mortality risk only**	**Associated with DAA treatment selection and mortality risk**	**Comments**
Age		√	Increased age is associated with increased mortality [3]. Age is a variable in APACHE II [19] and SAPS II [26]; also known that DAA typically given to younger patients [18-23]
APACHE II or SAPS II		**√**	Both are proven predictive mortality scores; DAA typically given to patients with higher scores [18-23]
Cardiovascular organ dysfunction		**√**	Mortality is higher in patients with septic shock versus sepsis without shock; DAA believed to be particularly effective in patients with shock [27]
Respiratory organ dysfunction	√		Respiratory dysfunction increases mortality [28,29]
Renal organ dysfunction	√		Renal dysfunction increases mortality [30,31]
Hematologic organ dysfunction		**√**	Hematologic dysfunction increases mortality [32,33]; DAA is particularly effective in patients with coagulopathy disorders (low platelets) [27]
Use of mechanical ventilation	√		Need for mechanical ventilation increases mortality [28,29]
Medical or surgical status		**√**	Type of admission is a variable in APACHE II and SAPS II [34,35]; recent surgery is a relative contraindication for DAA due to increased bleeding risk [7]
Site of primary infection		**√**	Predicted mortality varies with site of primary infection, but this is mostly a DAA selection bias variable; DAA may be particularly helpful when lung is source of primary infection [36]

The propensity score will be estimated using a logistic regression model for treatment group using the matching variables included in the calculation of Mahalanobis distances across all centers, plus a categorical variable for center. We will test for interaction between age and APACHE II or SAPS II scores and for interactions between age and each of the four organ dysfunctions (cardiovascular, respiratory, hematologic, and renal). If individual interactions are significant at the 0.05 level, then these interaction terms will be included in the propensity score model.

Calipers will be applied to selected key variables to ensure close matches. For age, a maximum 5-year difference was chosen based on clinical judgment of what seems “close” and with consideration of how age was handled in the APACHE II scoring system, which assigned age points based on 10-year intervals [34]. Thus, the 5-year caliper is tighter than the intervals used for calculating APACHE II scores.

For APACHE II scores, we expect scores to be predominantly in the range from 20–40; patients must be within 2 points of each other. A 2-point difference in APACHE II scores in this range would give a difference in predicted mortality of approximately 7% at the low end and 1% at the high end. In the original APACHE II publication [34], a 5-point difference in APACHE II scores was associated with a statistically significant difference in mortality risk. Therefore the two-point caliper for APACHE II is tighter than the difference that was statistically significant. For SAPS II, a four-point caliper will be applied to achieve comparability with the two-point caliper for APACHE II score [35].

The propensity score caliper will be set at the value that represents 0.6 standard deviations (of the average propensity score). This will define subgroups of approximately 20% of the sample within which a match must be made. Cochran and Rubin found that this leads to excellent bias reduction [37-39].

No imputation of missing data will be done to satisfy eligibility criteria. To support selection of matched patients (once deemed eligible for study), missing data for up to two matching variables will be allowed to be imputed for an individual patient. Missing data will be imputed using available data from that same center if for any matching variable, the proportion of missing values per center is <30% for the INDICATED population; if higher, imputation will not be done. No missing data imputations are allowed for age (an eligibility criterion) and for APACHE II or SAPS II scores due to their complexity.

A clinical research organization, Syreon Corporation, will conduct the study. A two-phase transfer of data from each center will be implemented to ensure that the selection of matched control patients is implemented in a blinded and unbiased manner. Data transfer 1 will include all variables needed to confirm eligibility and to conduct the matching. Once the matching has been completed, the matched sets of treated and control patients will be “locked” together. Then data transfer 2 (outcomes and genotypic data) will be sent to Syreon.

### Genotyping

Most centers will have already extracted DNA using standard techniques. Genotyping for the IRP SNPs will be done using a validated Taqman®-based analytical method, and the laboratory will be blinded to treatment and outcome. The plate layout of DNA samples for genotyping will be randomized to avoid systematic bias introduced from laboratory method conditions. Specific DAA-treated patients and their matched controls will be assigned to the same plate to ensure the tightest control of external factors within each set of matched patients. A panel of 93 Ancestry Informative Marker (AIM) SNPs will be genotyped using the Illumina GoldenGate® analytical method [38]. This method for ancestry assignments (using the STRUCTURE software package) has been shown to adequately identify patients of European, African, and Asian ancestry [40,41], the relevant ancestral groups for this study.

### Statistical analysis

The target sample size is >700 DAA-treated patients in the INDICATED population. If 750 DAA-treated patients were enrolled and approximately 1,500 matched control patients identified, this trial would have adequate power when testing two hypotheses, IRP A and IRP B, corrected for multiplicity testing. The study would have approximately 90% power to detect a treatment-by-IRP interaction based on an absolute reduction in mortality of 15% in the DAA-treated group compared with the control group in IRP + patients and with 1% to 2% difference in mortality between the treated and control groups in the IRP − patients.

The primary analysis will be conducted using the Matched-INDICATED population to compare the effect of treatment in the IRP + and IRP- groups by testing for the effect of the interaction between IRP and DAA treatment on the primary endpoint in a conditional logistic regression model, conditioning on the matching and incorporating the principal component scores from the AIM panel data as covariates to control for potential population stratification. The primary endpoint is in-hospital mortality through day 28 (i.e., patients are followed until hospital discharge or day 28, whichever comes first). Each of the primary analyses, one for IRP A and one for IRP B, will be conducted as a two-sided test with α = 2.5% for an overall, Bonferroni-corrected, type I error rate of 5%.

Estimates of the effect of treatment within each IRP status subgroup also will be provided as odds ratios and their 95% CIs from the conditional logistic regression analysis. For descriptive purposes, ARRs for each IRP status subgroup and their 95% CIs based on weighted mortality estimates also will be provided. Secondary analyses will include matching variables as covariates in the regression model to adjust for residual imbalances and possible confounding. Additionally, an ethnicity subgroup analysis will investigate the three-way interaction among ethnicity, treatment, and IRP in a matched conditional logistic regression model.

For secondary endpoints, stratified Cox regression will be used to estimate time to death in hospital (censored at discharge) and time to death (censored at day 60). Conditional logistic regression will be used to estimate the log-odds of mortality as a function of IRP, treatment group, interaction between IRP and treatment, conditioned on the matched sets. Mechanical ventilator-free days, ICU-free days, and hospital-free days (all through day 28) will be analyzed using Poisson regression models. The same analyses will be performed using the matched non-INDICATED population.

## Conclusions

A design-based approach matching DAA-free controls to DAA-treated patients in a multicenter study of patients who have severe sepsis and high risk of death decreases lack of balance between groups for variables associated with risk of death and response to DAA treatment. The matched control group tightens control of key variables associated with mortality and DAA treatment selection. This design-based strategy optimizes direct comparison of the control to the DAA-treated group for mortality by genotype. The matching and genotyping will be done blinded to outcomes. The sample size and power are adequate (>700 DAA-treated patients). The matched control patients will be selected from approximately 18,000 potential controls. This large ratio of DAA-free to DAA-treated patients assures good matching. Finally, the individual SNPs of the primary hypothesis IRPs were selected based on a GWAS in the pivotal PROWESS trial and alignment of strength and direction of signal in a replication cohort.

Because DAA use has been relatively low in the study cohorts, this type of matched-patients study can be conducted. If DAA were administered to most eligible patients, it would be difficult to find appropriate DAA-free patients to use as matched controls. DAA was typically used in less than 10% of the indicated patients across the cohorts.

To validate a genomic biomarker that can identify a subgroup of patients who would have an enriched DAA treatment effect, our goal is to find a *prescriptive* genomic biomarker that can guide the decision to treat with DAA. Such a biomarker would provide a strong rationale for reconsidering the use of DAA in a selected population of patients with severe sepsis. Moreover, this study will provide a unique model for future investigations that seek to identify subpopulations who respond to sepsis therapies.

## Competing interests

Dr. Russell reports holding stock in Sirius Genomics Incorporated, which has submitted patents owned by the University of British Columbia (UBC) and licensed to Sirius Genomics, which are related to the genetics of sepsis and its treatment. The University of British Columbia also has submitted a patent related to the use of vasopressin in septic shock. Drs. Russell and Gordon report being inventors on these patents. Dr. Russell reports receiving consulting fees from Ferring Pharmaceuticals (which manufactures vasopressin and is developing a selective V1a agonist), from Astra Zeneca (which is developing an anti-TNFα), from BioCritica (which used to sell activated protein C in the United States), and from Sirius Genomics Inc. Dr. Russell reports having received grant support from Sirius Genomics, Ferring Pharmaceuticals, Astra Zeneca, and Eli Lilly, which is provided to and administered by UBC. Dr. Russell has received speaking honoraria from Pfizer and Eli Lilly. Dr. Gordon has received consulting and speaker fees from Eli Lilly. Dr. Gordon reports having previously been employed by Sirius Genomics and subsequently receiving consulting fees.

## Authors’ contribution

DA, AM, and JR contributed to the conception and design of the study and drafted this manuscript. JPM, LW, AG, JS, and FS contributed to the design of the study and helped drafting the manuscript. PH, HW, MN contributed in the design of the study and developed the statistical plan. All authors read and approved the final manuscript.
